# Assessment of newborn neuropsychomotor development born with exposure to SARS-CoV-2 in the perinatal period using the Bayley III scale at 6 months of age

**DOI:** 10.1016/j.clinsp.2024.100460

**Published:** 2024-08-01

**Authors:** Patricia Albertini Orioli, Cintia Johnston, Juliana Zoboli Del Bigio, Vera Lucia Jornada Krebs, Mariana Pissolato, Maria Augusta Bento Cicaroni Gibelli, Orlei Ribeiro De Araujo, Rossana Pulcineli Vieira Francisco, Werther Brunow De Carvalho

**Affiliations:** a Department of Pediatrics, Faculdade de Medicina da Universidade de São Paulo (FMUSP), São Paulo, SP, Brasil; b Postgraduate Program in Pediatrics, Department of Pediatrics, Faculdade de Medicina da Universidade de São Paulo (FMUSP), São Paulo, SP, Brasil; c Faculdade de Medicina da Universidade de São Paulo (FMUSP), São Paulo, SP, Brasil; d Rede D´OR, Faculdade de Medicina da Universidade de São Paulo (FMUSP), São Paulo, SP, Brasil; e Institute of Pediatric Oncology, Support Group for Adolescents and Children with Cancer (GRAACC), Universidade Federal de São Paulo (UNIFESP), São Paulo, SP, Brazil; f Department of Obstetrics, Faculdade de Medicina da Universidade de São Paulo (FMUSP), São Paulo, SP, Brasil; g Neonatal Medicine and Intensive Care, Department of Pediatrics, Faculdade de Medicina da Universidade de São Paulo (FMUSP), São Paulo, SP, Brasil

**Keywords:** NPMD, Newborn, SARS-CoV-2, COVID-19, Bayley, Bayley-III

## Abstract

**Objective:**

This study aimed to evaluate the Neuropsychomotor Development (NPMD) of newborns exposed to SARS-CoV-2 in the perinatal period using the Bayley III scale at 6 months of age.

**Methods:**

Childcare appointments were scheduled for the included newborns in the study. During the 6-month consultation, the Screening Test for Bayley III Scale and, based on it, children were classified as “low risk”, “moderate risk” or “high risk” in the domains: of cognitive, receptive language, expressive language, fine motor, and gross motor. Those classified as “moderate risk”; or “high risk” received guidance about NPMD stimuli and were instructed to maintain follow-up.

**Results:**

Only 13 (37.1 %) of the newborns were classified as low risk in receptive language and 18 (51.4 %) in gross motor skills, with the domains most affected. Prematurity was a risk for cognitive incompetence (moderate risk/high-risk classification) (coefficient: 1.89, Odds Ratio = 6.7, 95 % CI 1.3‒35, p = 0.02). Lower birth weight that 2.500g had a similar effect on cognitive incompetence (coefficient: 1.9, Odds Ratio = 6.2, 95 % CI 1.2‒32.2, p = 0.02). Exclusive breastfeeding at hospital discharge (n = 8) was protective for incompetence (high risk/moderate risk) in the language domain (coefficient -2.14, OR = 0.12, 95 % CI 0.02‒0.71, p = 0.02).

**Conclusions:**

The children included in the study must be monitored and their development monitored in order to clarify whether there is a relationship between the delay in NPMD and perinatal exposure to COVID-19, as delays were observed in these preliminary results.

## Highlights

• The perinatal repercussions of the SARS-CoV-2 virus have not yet been elucidated.
• Few reports in the literature indicate damage to the Neuro Psychomotor Development (NPMD) of children born during the COVID-19 pandemic, but its mechanisms are not clearly established.

## Introduction

The repercussions of the infection caused by SARS-CoV-2 on pregnant women and newborns still remain unclear regarding their manifestations in the mother-child binomial.^1^, ^2^, ^3^ It is estimated that more than 200 million births have occurred since the beginning of the SARS-CoV-2 pandemic and, even the most conservative estimates, calculate that millions of babies around the world were exposed to the virus during pregnancy during this period.^4^

Transmission of SARS-CoV-2 occurs mainly through droplets from infected people or through contact with contaminated biological material. The spectrum of disease resulting from SARS-CoV-2 infection is called COVID-19 and can range from asymptomatic infection to pneumonia, which can lead to acute ventilatory failure, septic shock, organ failure, and death. SARS-CoV-2 is also a virus known for neurological complications due to its ability to directly damage neuronal cells in the cortex and hypothalamus.^4^, ^5^, ^6^, ^7^, ^8^

Regarding the vertical transmission of SARS-CoV-2 from mother to fetus, although rare and not completely understood, previous outbreaks of other coronavirus variants and exposures to other viruses (including the Human Immunodeficiency Virus ‒ HIV) suggest that serious infections during pregnancy may be associated with both risks to maternal health and a greater number of adverse outcomes in the newborn.^4^

These factors contributed to the hypothesis that newborns of mothers infected with SARS-CoV-2 during pregnancy may have a greater risk of stillbirth, prematurity, brain damage in the fetus and consequent changes in Neuropsychomotor Development (NPMD) due to the known ability of this virus to damage nerve cells.^4^^,^^7^ To date, there are still few reports in the literature about the impact of SARS-CoV-2 on the neuropsychomotor development of newborns exposed to SARS-CoV-2.^4^^,^^8^, ^9^, ^10^

Deoni SCN et al. (2021) found that COVID-19 changed the landscape of child health, as children born between 2020 and 2021 were placed in an economic, psychosocial and educational environment different from that which existed prior to the COVID-19 pandemic.^9^ Preliminary results showed significant evidence of reductions in cognitive function and motor performance in children born during the pandemic.^9^^,^^10^

Shuffrey LC et al. (2022) also evaluated the NPMD of babies exposed to SARS-COV-2 in the intrauterine environment, but their results showed that exposure to maternal virus infection was not associated with development damage.^4^ However, compared to the historical cohort, newborns during the pandemic had lower scores in the gross motor, fine motor, and personal-social domains.

In the literature, there are several instruments designed to assess child development, all of which have their advantages and limitations.^11^ The Bayley III Child Development Scales assess children between one and 42 months of age and have a screening test, The Bayley Infant Neurodevelopment Screener (BINS), which identifies possible delays in the NPMD and indicates whether a more extensive evaluation is necessary.^11^, ^12^, ^13^, ^14^, ^15^

Orioli PA et al. (2022) showed in a systematic review that despite presenting a sensitivity of 33.3 %, specificity of 98 % and level of evidence C, the Bayley III Scales can be used to monitor gains after interventions.^14^ This level of evidence is due to few studies and small samples when applying this Scale, but it is a tool validated in the Brazilian population, with standardized language, which can be applied by trained health professionals and duly certified for this purpose, being considered gold standard.^1^^,^^11^^,^^14^

Knowledge of the NPMD of newborns exposed to SARS-CoV-2 through a specific scale validated for the Brazilian population is important for the detection and prevention of possible functional changes.^4^^,^^8^, ^9^, ^10^ Monitoring children born during the COVID-19 pandemic is necessary for the early identification of children at risk for delays in NPMD.

Thus, the objective of this study was to describe the maternal and newborn characteristics and evaluate the NPMD of those exposed to SARS-CoV-2 in the perinatal period using the Bayley III Scale Screening Test at six months of chronological age, aiming to identify possible changes in the motor, cognitive and language domains.

## Methods

This is a prospective unicentric study, including newborns at the Hospital das Clínicas of the Faculty of Medicine of USP (HCFMUSP) and admitted to the Neonatal Intensive Care Center (CTIN1) of the Children's Institute of FMUSP from to 04/26/2020 on 2/17/21.

Newborns whose mothers had flu-like syndrome and RT-PCR (at the time of birth) for SARS-CoV-2 positive and/or positive serology during the gestational period were included.

Newborns whose birth took place in external institutions, newborns with major congenital malformations (examples: complex heart diseases, genetic syndromes, known alterations of the nervous system), or when the legal guardian's acceptance to participate in the research were not obtained.

For the children included in the study, childcare consultations were scheduled at the Neonatology Outpatient Clinic of the HCFMUSP Children's Institute, so that an assessment was mandatory at 6 months of chronological age.

During the consultations, the Bayley III Scale Screening Test^12^, ^13^, ^14^, ^15^ was applied. From there, the children were classified as “low risk”, “moderate risk” or “high risk” in each of the domain assessments: cognitive, receptive language, expressive language, fine motor, and gross motor. Those classified as “moderate risk”, or “high risk” received guidance on NPMD incentives and were advised to maintain follow-up to carry out the assessment in the following months.

The assessments were carried out by two pediatricians and neonatologists previously trained and qualified to apply and interpret the results of the Bayley III Scale and reviewed by a physiotherapist qualified to use these assessment scales.

The study received approval to be carried out by the Research Ethics Committee: CAAE: 44944820.3.0000.0068; CAPPESQ Approval ‒ Opinion n° 4,764,713.

### Statistical analysis

The data were tabulated in the Excel 2019 program (Microsoft Corporation, USA). Descriptive statistics used measures of central dispersion (means and Standard Deviations [SD], medians and Interquartile Ranges – IIQ: 25 %‒75 % percentiles), as well as absolute and relative frequencies, as indicated.

The analyses were performed using R: A language and environment for statistical computing. Version 4.3.0. R Foundation for Statistical Computing, Vienna, Austria. URLhttps://www.R-project.org/.

The variables tested in the bivariate models were prematurity, length of stay, birth weight, antibiotics, parenteral nutrition, resuscitation, Apgar in the 1^st^ and 5^th^ minute, acute fetal distress and type of delivery.

Bivariate regression models were constructed for the outcomes: cognitive competence, in receptive language, expressive language, fine and gross motor skills, at six months of chronological age.

## Results

**Fig. 1:**
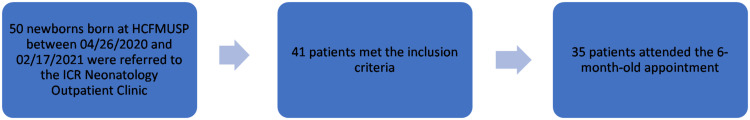
Description of newborns in the period, those with inclusion criteria and those who appeared for evaluation by the Bayley III Screening Test.

During the period of this unprecedented study, 50 babies were born at the Institution, 41 of which met the study inclusion criteria. However, 35 patients were included in the study (Fig. 1) due to the difficulties imposed by the beginning of the pandemic, such as mobility restrictions, socioeconomic issues and distance logistics for patients who were referred to a tertiary center far from their residence at the time of birth.

Data from 41 patients were analyzed. Maternal epidemiological data and birth conditions are found in Table 1.

**Table 1:** Maternal data and birth conditions.

Type of birth	n	%
Cesarean delivery	33	80.5 %
Vaginal birth	7	17.1 %
Forceps	1	2.4 %
**Indication for cesarean delivery**		
COVID-19	14	34.1 %
Oligohydramnios	5	12.2 %
Acute fetal distress	5	12.2 %
Dystocia	4	9.8 %
Broken bag	2	4.9 %
Other maternal causes (Hypertensive disorders of pregnancy/age)	4	9.8 %
**Moment of maternal COVID-19**		
Delivery or up to 14 days before delivery	32	78.0 %
First trimester of pregnancy (up to 13 weeks)	2	4.9 %
Second trimester of pregnancy (up to 27 weeks)	5	12.2 %
Third trimester of pregnancy (greater than or equal to 28 weeks and more than 14 days from birth)	2	4.9 %
**Mother's PCR COVID-19**	41	100 %
Positive at birth	33	80.5 %
**Maternal outcomes**		
Mother's intubation	4	9.8 %
Maternal death	4	9.8 %
**Housing conditions**		
Basic sanitation	40	97.5 %
Number of rooms in the house (mean, SD)	3.7	1.8
Number of residents in the house (mean, SD)	3.8	1.4

Data on the birth conditions and evolution during hospitalization of the newborns can be seen in Table 2. As in Table 3, complementary exams carried out on the newborns throughout the period of hospital stay and outpatient follow-up are shown.

**Table 2:** Data and clinical evolution of newborns.

**Gender**		
Girls	20	48.8 %
Boys	21	51.2 %
**Gestational age**		
< 28 weeks	3	7.3 %
From 28 weeks until 31 weeks e 6 days	3	7.3 %
From 32 weeks until 33 weeks e 6 days	5	12.2 %
From 34 weeks until 36 weeks e 6 days	8	19.5 %
≥ 37 weeks	22	53.7 %
**Birth weight**		
Birth weight (grams, mean)	2651	845.7
Fenton weight (median, IIQ)	51 %	26‒63 %
Maximum weight (g)	3870	
Minimum weight (g)	680	
**Birth conditions**		
Apgar 1^st^ min (median, IIQ)	8	7‒9
Apgar 5^th^ min (median, IIQ)	9	9‒9
Need for resuscitation, n (%)	7	17.1 %
**Clinical evolution of the newborn**		
Length of stay (mediana, IIQ)	4	5.5‒13
Vasoactive medications	2	4.9 %
Antibiotics	8	19.5 %
Parenteral nutrition	7	17.1 %
Phototherapy	15	36.6 %
**Diet at discharge**		
Breast milk	8	19.5 %
Artificial formula	30	73.2 %
Mixed breastfeeding	3	7.3 %
**COVID-19 investigation in newborn**		
Positive PCR for COVID-19 in newborn	2	4.9 %
RN serology age (months, median, IIQ)	4	2‒5
Positive igG in newborn (N36)	6	16.6 %

PCR, Polymerase Chair Reaction test; IGG, Immunoglobulin G; IIQ, Interquartile Range.

**Table 3:** Newborns’ complementary exams.

**USG skull**	32
USG skull findings:	
Normal	26
Peri-intraventricular hemorrhage	2
Choroid plexus/thalamus/ependyma cysts	3
Calcifications in the thalamus	1
**BAEP** (Brainstem Auditory Evoked Potential)	31
Normal BAEP	31
**Eye fundus examination**	20
Fundus examination findings:	
Normal	16
VI Z II (incomplete vascularization zone II without ROP)	2
VI ZIII (incomplete vascularization zone III without ROP)	2
**Echocardiogram**	28
Normal	11
Patent foramen ovale	11
Interatrial communication	4
Interatrial communication + patent ductus arteriosus	1
Patent foramen ovale + interventricular communication	1

USG, Ultrasound; ROP, Retinopathy of Prematurity.

Table 4 shows the assessment of the NPMD of newborns at six months of chronological age, carried out using the Bayley III Scale Screening Test. The newborns were evaluated in the cognitive, receptive language, expressive language, fine motor and gross motor domains and classified according to the score achieved as: “low risk”, “moderate risk” and “high risk”.

**Table 4:** Bayley III Scale Screening Test applied at 6-months of chronological age of newborns.

	**Cognitive score**			
**Low risk**	26	74.3 %		
**Moderate risk**	8	22.9 %		
**High risk**	1	2.9 %		
**N**	35			
	**Receptive language score**		**Expressive language score**	
**Low risk**	13	37.1 %	24	68.6 %
**Moderate risk**	18	51.4 %	8	22.9 %
**High risk**	4	11.4 %	3	8.6 %
**N**	35		35	
	**Fine motor score**		**Gross motor score**	
**Low risk**	20	57.1 %	18	51.4 %
**Moderate risk**	11	31.4 %	13	37.1 %
**High risk**	4	11.4 %	4	11.4 %
**N**	35		35	

**Fig. 2:**
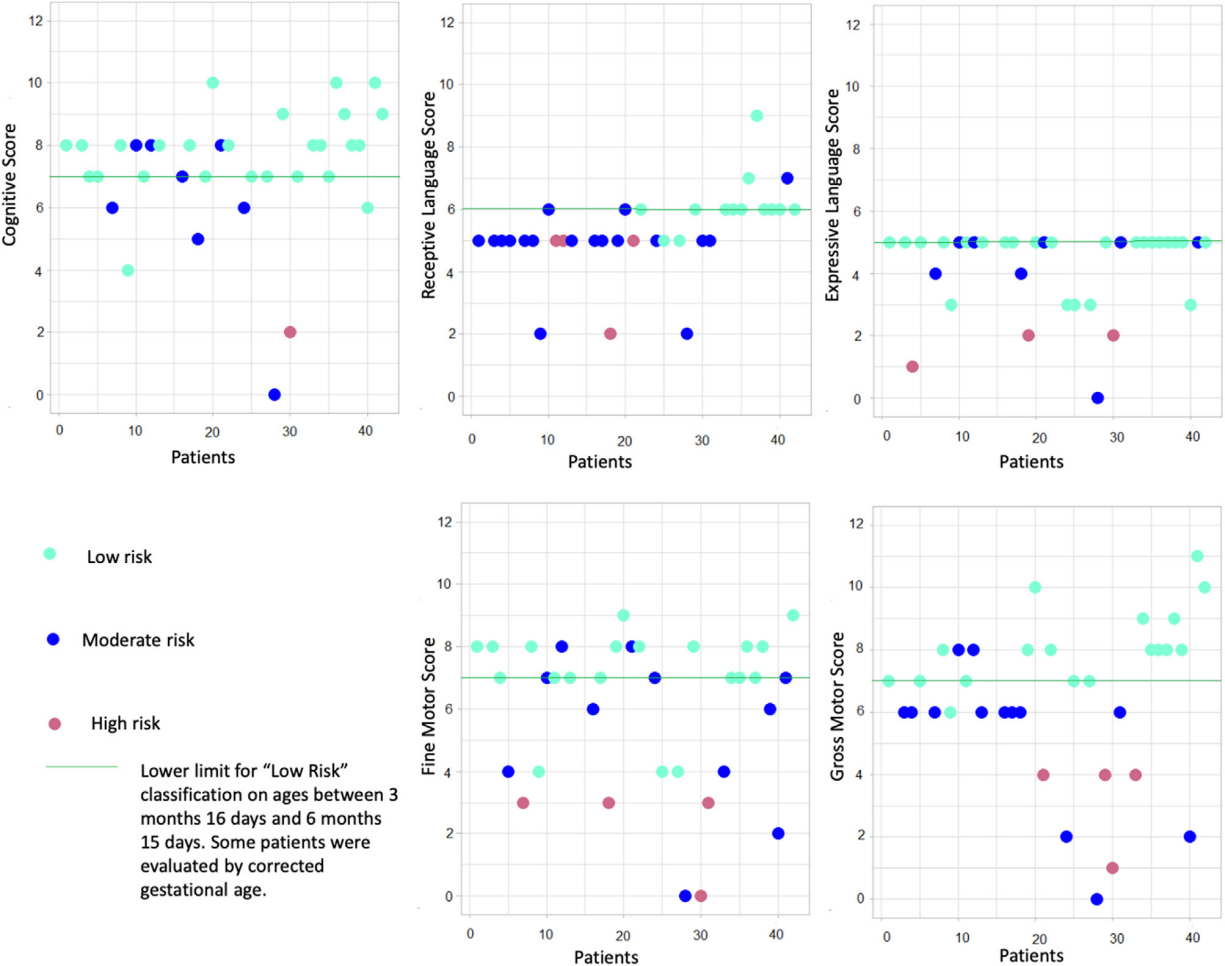
Scatter plot of the Screening Test classifications in the domains assessed at six months of age.

Figure 2 shows the scatter plot in which it is possible to observe the classifications achieved by the evaluated patients (low risk / moderate risk / high risk) in the five domains evaluated (cognitive / receptive language / expressive language / fine motor / gross motor) at six months of age.

Prematurity (gestational age < 36 weeks) was a risk for cognitive incompetence (coefficient: 1.89, Odds Ratio 6.7, 95 % CI: 1.3‒35, p = 0.02). Birth weight (< 2500g) showed a similar effect for cognitive incompetence (coefficient: 1.9, Odds Ratio: 6.2, 95 % CI: 1.2‒32.2, p = 0.02).

Exclusive breastfeeding at hospital discharge (n = 8) was protective against incompetence (high risk/ moderate risk) in the receptive language domain at six months of age (coefficient: -2.14, OR = 0.12, 95 % CI: 0.02‒0.71, p = 0.02).

For all other variables in binary combinations with the outcomes, there were no significant risks.

## Discussion

Of the 41 mothers whose children were included in the study, four required orotracheal intubation at the time of birth (9.8 %), two of whom died. The maternal mortality rate in this study was 9.8 %, as four mothers died during hospitalization. Of these six mothers (14.5 % of the total) with the worst clinical evolution (orotracheal intubation and/or death), all had a cesarean section, in four of them there was an indication to interrupt the pregnancy due to severe COVID-19, one of them presented acute fetal distress and decompensation of hypertensive disorders of pregnancy.

Pregnant women are at greater risk of acquiring viral respiratory infections and developing serious clinical conditions due to the physiological, immunological and cardiopulmonary changes inherent to pregnancy.^1^^,^^6^^,^^16^^,^^17^ The information in the literature on this issue is still conflicting. While some point out that the clinical evolution of pregnant women with COVID-19 was similar to that of adults with COVID-19,^1^^,^^16^ other authors suggest that pregnant women may have worse outcomes, including eclampsia, admission to the Intensive Care Unit (ICU), use of mechanical ventilation and increased morbidity and mortality.^6^^,^^17^^,^^18^

The premature birth rate in the present study sample was 46.3 % and probably reflects the effects of COVID-19 on maternal health. Among 19 premature births, 17 were cesarean sections (89.45 %) and the study cesarean rate was 80.5 %. The literature states that COVID-19 during pregnancy was not associated with spontaneous premature birth.^16^ However, depending on the evolution of maternal respiratory failure, hypoxemia can alter the placental flow,^17^ contributing to the pregnancy being interrupted before full-term birth (before 37 weeks of gestation). Another study[16] points out that the prematurity rate was also influenced by issues unrelated to COVID-19, such as severe pre-eclampsia and premature rupture of ovular membranes.^16^

The lowest birth weight among the patients included in the sample was 680g, with an average weight of 2,651g. The median weight curve in the Fenton table was 51 %. Only two newborns were classified as small for gestational age (4.9 %), that is, weighing less than the 10^th^ percentile curve of the Fenton weight table. Apparently, in this sample, COVID-19 was not a risk factor for intrauterine growth restriction, a result also found in other studies.^1^^,^^16^^,^^18^

Regarding birth conditions, more than 80 % of the sample (82.9 %) did not require resuscitation maneuvers. Four patients (9.7 %) had an APGAR between 0 and 3 in the first minute and of these, 2 (4.8 %) remained with an APGAR lower than or equal to 3 in the fifth minute of life, therefore being classified as severely asphyxiated.

Of the total number of newborns, only 5 (12.1 %) had a fifth-minute APGAR score lower than 7. The sample, therefore, was predominantly composed of newborns who did not require resuscitation and did not suffer from perinatal asphyxia/depression, which could increase the risk of delays in the NPMD.

The absence of high rates of perinatal asphyxia related to COVID-19 is similar to other studies including pregnant women with COVID-19, which did not present newborns with a higher risk of perinatal asphyxia.^1^^,^^16^^,^^18^

Of the 41 newborns included in the study, 2 (4.8 %) were positive in the PCR for COVID-19, collected in the maternity ward.

Subsequently, during outpatient follow-up, IgG for COVID-19 was collected from 36 patients, with an average age of three to four months of age. Of these, 6 (16.6 %) showed reactive IgG. Among those with positive PCR initially, both also had positive serologies. The data available in the literature to date highlight that transplacental transmission remains controversial.^1^^,^^16^^,^^17^^,^^19^ Studies 1 highlights that although possible, it is apparently rare and unlikely.^6^^,^^20^

Regarding the diagnostic methods used, in the present study, two newborns presented positive PCR (collected upon admission) and serology (IgG, collected on an outpatient basis). However, it is not possible to prove transplacental transmission, as in most available studies, due to the high possibility of neonatal transmission due to possible failures in adequate isolation.^6^^,^^16^^,^^19^^,^^21^

At the time of discharge, only eight newborns (19.5 %) were exclusively breastfed, 30 (73.2 %) were artificially breastfed and 3 (7.3 %) were mixed breastfed. This low rate of breastfeeding reflected the need to accommodate newborns separately from their mothers, during hospitalization during the COVID-19 pandemic, following institutional protocol.

At six months of age, the exclusive breastfeeding rate was 31.7 % (13 patients). Exclusive breastfeeding at hospital discharge (n = 8) was protective against incompetence (high risk/I moderate risk) in the receptive language domain at six months of age (coefficient: -2.14, OR = 0.12, 95 % CI: 0.02‒0.71, p = 0.02).

### Bayley III screening test

Thirty-five infants were evaluated for MPND using the Bayley III Scale Screening Test when they were approximately six months of chronological age.

Aiming to minimize the effects of prematurity on the NPMD assessment, all premature infants included were evaluated considering their corrected gestational age. In this way, the 12 premature babies evaluated had their results based on their corrected ages, allowing them to be evaluated according to the expected acquisitions for their corrected age and minimizing the bias that prematurity could possibly represent in the detection of PMND delay.

In a recent study, it was suggested that despite showing a tendency to lower scores at 6-months of age, premature babies show greater gains in cognitive scores throughout the first year of life, suggesting a capacity for recovery despite restrictions imposed by premature birth.^22^

In the cognitive analysis of the 35 infants, 26 of them were classified as low risk (74.3 %), eight were classified as moderate risk (22.9 %) and 1 (2.9 %) as high risk for cognitive delay.

Language assessment was subdivided into receptive and expressive language. Only 13 infants (37.1 %) were classified as low risk in receptive language, 18 (51.4 %) were classified as moderate risk and 4 (11.4 %) as high risk. Thus, receptive language was the most affected area in the present study, as the majority of newborns presented cognitive incompetence (it was classified as “moderate risk” or “high risk”).

In the expressive language section, 24 children (68.6 %) were classified as low risk, 8 (22.9 %) as moderate risk and 3 (8.6 %) as high risk.

The motor assessment was subdivided into fine and gross motor skills. In the fine motor assessment, 20 children (57.1 %) were classified as low risk, 11 (31.4 %) as moderate risk and 4 (11.4 %) as high risk. In the assessment of gross motor skills, only 18 infants (51.4 %) were classified as low risk, 13 (37.1 %) as moderate risk and 4 (11.4 %) as high risk, which configured gross motor skills as the second most affected area, as practically half of the RNs did not reach a score that would classify them as low risk in the domain.

The application of the Bayley III Scales Screening Test in this study suggests that there was damage to the NPMD in infants exposed to COVID-19 in the perinatal period. However, it is necessary to continue monitoring these newborns to assess whether these changes are transient, permanent, or evolutionary. Therefore, all infants included in the evaluation at six months of chronological age were to be monitored for up to one year of life at the institution's Neonatology Outpatient Clinic.

Among the limitations of the findings of the present study, the authors include the difficulty in finding the specific cause of the changes in the NPMD of the sample, as they may be due to the repercussions of maternal COVID-19 disease during the gestational period, the direct influence of the virus and/or be due to changes in the socioeconomic conditions of the population during the pandemic (example: reduction of motor stimuli, social isolation). The logistic regression analysis in this sample has limitations, as it is a small sample. Due to the dependency relationship between the significant variables (prematurity, low birth weight, length of hospital stay), multivariate analysis was not possible.

## Conclusion

The assessment of NPMD of newborns exposed to SARS-CoV-2 in the perinatal period using a specific scale validated for the Brazilian population is not described in the literature.

The sample in this study presented lower scores in the Receptive Language and Gross Motor domains in the Bayley III Scales screening test, which may suggest possible impairment of NPMD in children exposed to SARS-CoV-2 in the perinatal period.

Infants included in the study must be monitored and their development monitored in order to try to clarify whether changes in NPMD are permanent, transitory or evolutionary.

## Declaration of competing interest

The authors declare no conflicts of interest.
